# Accuracy of Image Guidance Using Free-Breathing Cone-Beam Computed Tomography for Stereotactic Lung Radiotherapy

**DOI:** 10.1371/journal.pone.0126152

**Published:** 2015-05-08

**Authors:** Takeshi Kamomae, Hajime Monzen, Shinichi Nakayama, Rika Mizote, Yuuichi Oonishi, Soichiro Kaneshige, Takashi Sakamoto

**Affiliations:** 1 Department of Therapeutic Radiology, Nagoya University Graduate School of Medicine, Nagoya, Japan; 2 Department of Radiation Oncology, Okayama Central Hospital, Okayama, Japan; 3 Department of Radiation Oncology, Graduate School of Medical Science, Kinki University, Osaka, Japan; 4 Division of Clinical Radiology Service, Okayama Central Hospital, Okayama, Japan; University of Nebraska Medical Center, UNITED STATES

## Abstract

Movement of the target object during cone-beam computed tomography (CBCT) leads to motion blurring artifacts. The accuracy of manual image matching in image-guided radiotherapy depends on the image quality. We aimed to assess the accuracy of target position localization using free-breathing CBCT during stereotactic lung radiotherapy. The Vero4DRT linear accelerator device was used for the examinations. Reference point discrepancies between the MV X-ray beam and the CBCT system were calculated using a phantom device with a centrally mounted steel ball. The precision of manual image matching between the CBCT and the averaged intensity (AI) images restructured from four-dimensional CT (4DCT) was estimated with a respiratory motion phantom, as determined in evaluations by five independent operators. Reference point discrepancies between the MV X-ray beam and the CBCT image-guidance systems, categorized as left-right (LR), anterior-posterior (AP), and superior-inferior (SI), were 0.33 ± 0.09, 0.16 ± 0.07, and 0.05 ± 0.04 mm, respectively. The LR, AP, and SI values for residual errors from manual image matching were -0.03 ± 0.22, 0.07 ± 0.25, and -0.79 ± 0.68 mm, respectively. The accuracy of target position localization using the Vero4DRT system in our center was 1.07 ± 1.23 mm (2 SD). This study experimentally demonstrated the sufficient level of geometric accuracy using the free-breathing CBCT and the image-guidance system mounted on the Vero4DRT. However, the inter-observer variation and systematic localization error of image matching substantially affected the overall geometric accuracy. Therefore, when using the free-breathing CBCT images, careful consideration of image matching is especially important.

## Introduction

Stereotactic body radiation therapy (SBRT) has received favorable performance evaluations with respect to the treatment of early-stage lung tumors [1, 2]. The major features of SBRT are the fewer fractions and higher radiation doses per fraction than conventional radiation therapy. In order to minimize the normal tissue toxicity, conformation of high-dose region to the target and the steepness of the dose fall-off outside the target is critical [3, 4]. Therefore, in SBRT, high confidence level in the accuracy of target localization is required.

For intracranial tumors, radiotherapy is carefully administered to the precise tumor location within the skull. A pin brace is fixed directly to the skull with a gamma knife, to ensure accurate patient setup and target localization, and to facilitate margin reduction. For SBRT applied to the lungs, it is necessary to establish an internal target volume (ITV), to account for the movement of target due to factors such as respiration [5]. Moreover, suitable static anatomical information for target localization does not exist in the surrounding area; thus, when performing the target localization, careful consideration of unfixed structural information is necessary.

In recent years, image-guided radiotherapy (IGRT) has become available in a large number of clinics. IGRT assures high spatial accuracy by using a series of two-dimensional (2D) X-ray images, and CT images. Skeletal images serve as indices for position comparison when using an IGRT system with 2D X-ray images. On the other hand, when using an IGRT system with three-dimensional (3D) CT images, it is possible to use both bones and soft tissues as indices for position comparison. CT equipment in treatment rooms is categorized as kilovoltage (kV)-cone-beam computed tomography (CBCT), megavoltage (MV)-CBCT, and CT on-rails. CBCT imaging is conducted after half to full rotation of the gantry. Approximately 30–60 s of continuous image compilation is necessary for image completion.

CT images are acquired for use in treatment planning and reference patient positioning, where the X-ray tube and detector rotates faster than 1 s per revolution; thus, increased time resolution can be obtained. Moreover, CT equipment offers the potential for creating four-dimensional (4D) CT images by simultaneously obtaining topological phase data [6]. In contrast, CBCT requires long scan times, which results in lower time resolution compared with CT images.

Clear images of the contours and form of internal organs in areas subjected to minimal physiological motion, including the pelvis, head, and neck regions, can be obtained using CBCT. Further, by incorporating CT simulation images and CBCT images, it is possible to ensure geometrically precise treatment [7, 8]. However, CBCT requires a sufficiently long imaging time. Consequently, when CBCT imaging is conducted on a target subject to respiratory motion, image blurring occurs [9]. Thus, free-breathing CBCT images are known to exhibit a high affinity for averaged intensity (AI)-CT images restructured from a 4DCT dataset [10, 11]. However, based on the characteristics of the imaging equipment, the AI-CT and CBCT images are not completely identical [7]. As well, discrepancies between these images decrease with the accuracy of patient positioning.

**Fig 1:**
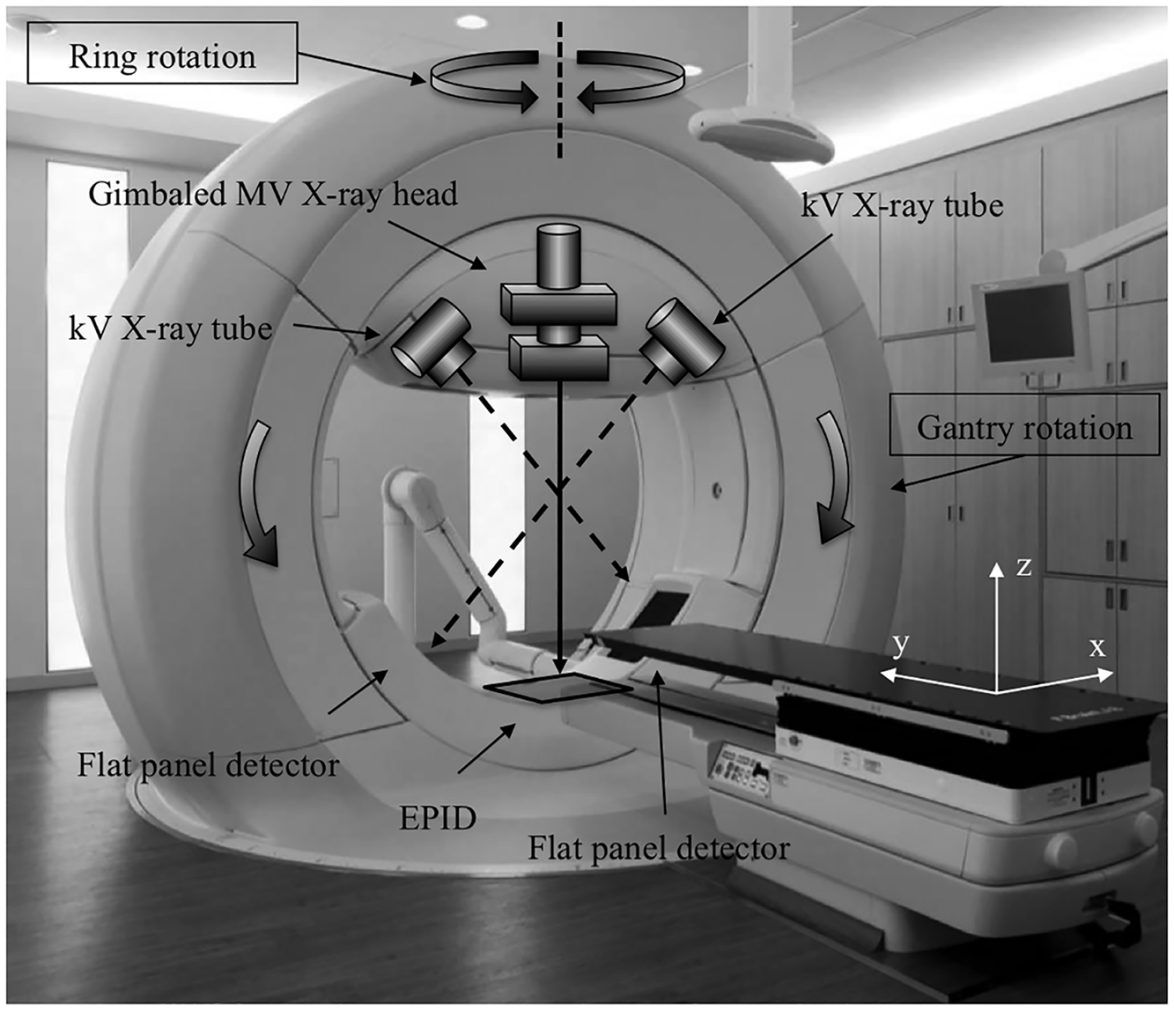
The Vero4DRT system with dual implementation of the kV imaging system and electronic portal imaging device (EPID). Exposure by non-coplanar MV beams was enabled by gantry and ring rotation. Dynamic tumor motion tracking was conducted through exposure to the gimbaled MV X-ray head. The lock-on system was used to correct sagging by the X-ray beam axis.

The Vero4DRT (MHI-TM2000, Mitsubishi Heavy Industries, Ltd., Japan, and BrainLAB, Feldkirchen, Germany) uses a linear accelerator component, equipped with both dynamic motion tracking and an IGRT system [12, 13]. The Vero4DRT unit is detailed in Fig 1. Dynamic tumor tracking is performed by swinging the MV X-ray head using the built-in gimbal mechanism [14, 15]. The Vero4DRT has the ability to correct any drift in the MV X-ray beam axis caused by its own weight, through the use of the gimbal mechanism, commonly referred to as the “lock-on system.” Compared with other commercial linear accelerators, we expected improved consistency of the beam axis using the lock-on system. Additionally, the Vero4DRT is equipped with a dual orthogonal kV imaging system. Similarly, the kV CBCT also has imaging capability. Accordingly, the Vero4DRT executes IGRT through the use of these two types of imaging systems [16].

The purposes of this study were to assess the overall geometric accuracy of the Vero4DRT CBCT system, as well as the accuracy of target position localization using the free-breathing CBCT images during SBRT. To achieve our objectives, we investigated the reference point discrepancies between the MV treatment beam and the CBCT image-guidance system, as well as the precision of manual image matching between the reference AI-CT images and the free-breathing CBCT images determined by the evaluations of five independent operators.

## Materials and Methods

### Coincidence of the CBCT image center and MV beam isocenter

**Fig 2:**
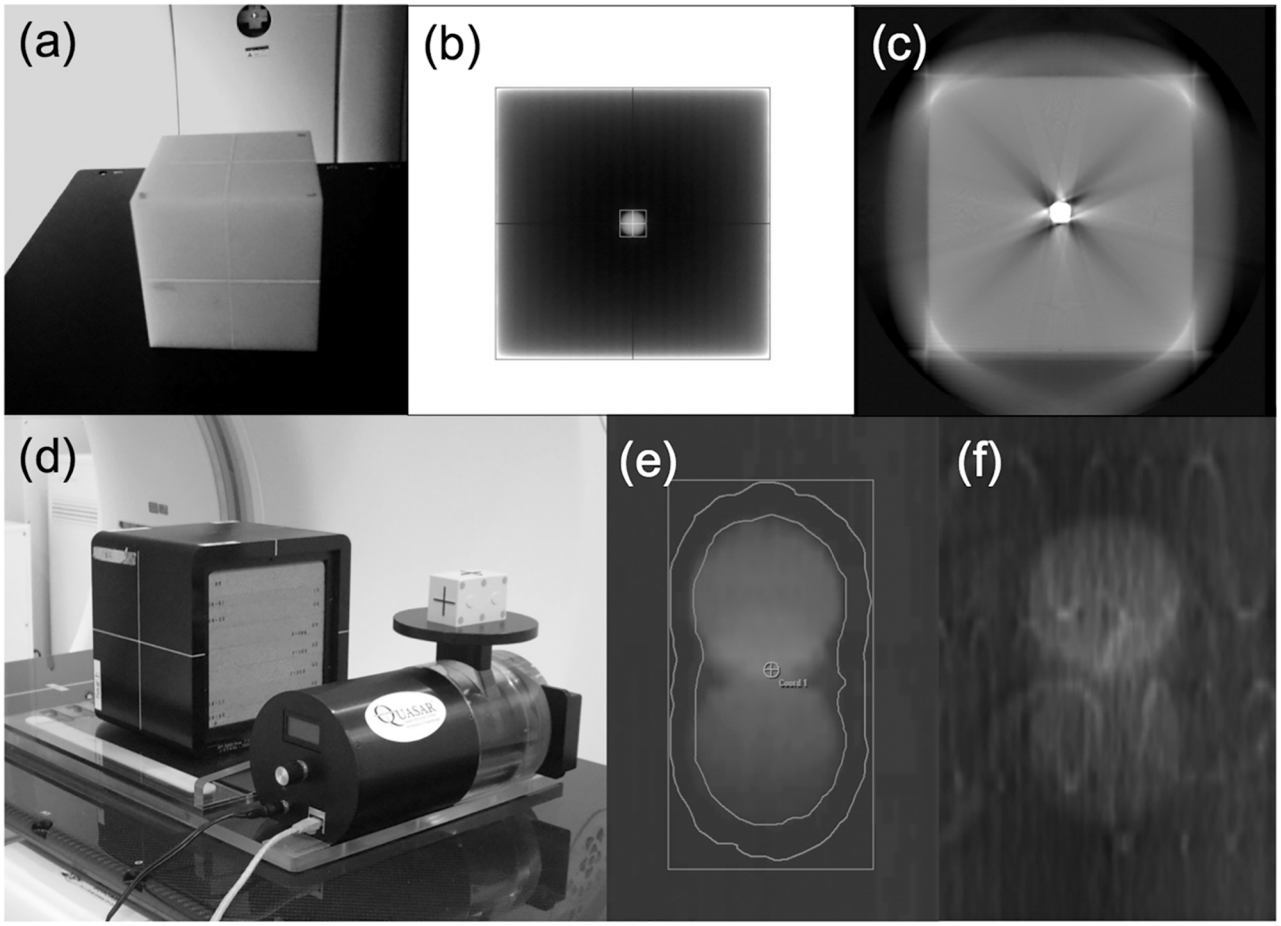
A phantom device with a centrally inserted steel ball was used to evaluate the reference point discrepancies between the MV beam and the CBCT system. Photographs of (a) the phantom exterior, (b) EPID image, and (c) CBCT image. The precision of manual image matching between the averaged intensity (AI)-CT and CBCT images was evaluated by the motion platform with the lung phantom. Photographs of (d) the phantom exterior, (e) the AI-CT image with contour information for the ITV and the PTV, and (f) the CBCT image.

We installed a steel-ball-equipped phantom device (Fig 2a) in the Vero4DRT unit. The radiation field was set to 100 × 100 mm^2^ MV X-ray irradiation. A visual representation of the X-ray penetration of the phantom device, provided by an electronic portal-imaging device (EPID), is shown in Fig 2b. The procedure was repeated for gantry angles of 0°, 90°, 180°, and 270°, and ring angles of 0°, 20°, and 340°. The 2D positioning discrepancy between the steel ball unit and the X-ray field from the projected EPID image was calculated using the Daily Check tool in the Vero4DRT unit. When the Vero4DRT unit was commissioned, we confirmed, using radiochromic films, that the concurrence of the results of the Daily Check tool and the Winston-Lutz tests were 0.02 ± 0.11 mm. Discrepancies between the steel ball unit and the X-ray field in the EPID image coordinates were then converted into a coordinates (x, y, z), as shown in Fig 1, corresponding to the patient coordinates: left-right (LR); superior-inferior (SI); and anterior-posterior (AP), respectively. These coordinate conversions were performed based on the parameters of gantry and ring rotation angle.

Fig 2c contains a sample of the CBCT images with respect to the steel-ball-equipped phantom. We obtained pattern data from various equipment and operation combinations, consisting of kV X-ray tubes 1 and 2, and with clockwise (CW) or counterclockwise (CCW) tube rotation. The 3D positioning discrepancy between the CBCT image center and MV beam isocenter was calculated using the following equations:

$$D_{MV}(x,y,z)=P_{ball}(x,y,z)-P_{MV}(x,y,z),$$ (1)

$$D_{CBCT}(x,y,z)=P_{ball}(x,y,z)-P_{CBCT}(x,y,z),$$ (2)

$$D_{CBCT-MV}(x,y,z)=D_{CBCT}(x,y,z)-D_{MV}(x,y,z),$$ (3)

where *D*_*MV*_ is the positional discrepancy between the MV X-ray beam and the steel ball. *P*_*ball*,_ and *P*_*MV*_ are the primary reference points for the steel ball and MV X-ray field, respectively. Further, *D*_*CBCT*_ is the positional discrepancy between the CBCT image and the steel ball, *P*_*CBCT*_ is the primary reference point for the CBCT image, and *D*_*CBCT-MV*_ is the discrepancy between the primary reference points for the CBCT image and the MV X-ray field. This procedure was repeated four times, which is representative of the four-day SBRT treatment delivery schedule [1].

### Manual image matching using blurred images for AI-CT and CBCT

A QUASAR programmable respiratory motion platform (Modus Medical Devices Inc., London, ON, Canada) and a lung phantom (RT-3000-New; R-TECH, Inc., Tokyo, Japan) were placed on the CT simulation system (OptimaCT580W; GE Healthcare Technologies, Waukesha, WI). Fig 2d shows a photograph of the motion platform and the lung phantom. The spherical objects embedded in the lung phantom (RT-3000-New) were a simulated tumor with a diameter of 30 mm (large simulated tumor) or 20 mm (small simulated tumor). To confirm the default positional discrepancies of the phantom between CT imaging and CBCT imaging, four small 2 mm diameter steel markers were embedded in the lung phantom. For the theoretical breathing pattern study, the respiratory cycle time, *T*, varied between 2.0 and 4.0 s, and the largest oscillation width, *A*_1_, ranged from 1.5 to 3.0 cm. The motion pattern was applied using the waveform model given below [17]:

$$A(t)=A_{0}-A_{1}\cos^{6}(\pi t/T-\phi )$$ (4)

where *A*(*t*) is the amplitude at time *t*, *A*_0_ is the baseline position, and *ϕ* is the initial phase. The respiratory waveform of 10 patients who underwent lung SBRT was used in the evaluation of the patient breathing patterns. The study was approved by the Institutional Review Board of the Okayama Central Hospital, and all patients provided written informed consent. The average patient amplitude was 5.0 ± 2.2 mm and the average respiratory cycle was 3.7 ± 1.0 s. We used the large simulated tumor to account for the cases in which the actual tumor diameter in the axial CT image was greater than 2.5 mm. On the other hand, we used the small simulated tumor to account for the cases in which the actual tumor diameter in the axial CT image was smaller than 2.5 mm. We conducted 4DCT imaging using the Real-time Positioning Management System (Varian Medical Systems, Palo Alto, CA). The imaging conditions were as follows: the tube voltage was 120 kV, the tube current was 100 mA, the X-ray tube rotation time was 1 s, the cine duration time of the scan in each couch position was 6 s, the field of view (FOV) was 500 mm, and the slice thickness was 2.5 mm. All images were imported into the Advantage 4-D workstation (GE Healthcare Technologies, Waukesha, WI), and the maximum intensity projection (MIP) images, and the AI-CT images were subsequently created. Next, the MIP and AI-CT images were imported into the treatment planning system (iPlan version 4.5; BrainLAB AG, Feldkirchen, Germany). The first step was to establish an internal treatment volume (ITV) from the MIP image. The planning target volume (PTV) was then obtained by adding an ITV margin of +5 mm [18]. Next, 18 treatment plans were created to evaluate the precision of manual image matching, by intentionally manipulating the isocenter by 0 to 10 mm from the original isocenter point. The ExacTrac system (BrainLAB AG, Feldkirchen, Germany) is integrated into the Vero4DRT system, and provides a patient-positioning function [16]. Accordingly, the treatment plan and the AI-CT images were transmitted to the ExacTrac system.

The phantom device was settled above the couch top in the Vero4DRT and aligned based on the visible markers on the phantom’s surface using the lasers. Reference CBCT images were acquired with the stationary phantom. The geometric misalignment between the AI-CT and CBCT images was estimated, and corrections were made using the four small steel markers placed in the phantom. Reference CBCT images were then reacquired and the residual positional error was confirmed. The residual positional error was the criteria used for the manual image matching performed in this study. The CBCT images were then acquired under the same moving conditions as described for 4DCT imaging. The CBCT imaging conditions were as follows: gantry rotation angle = 200°, imaging time = 29 s, FOV = 215 mm, and slice thickness = 2.0 mm.

The initial evaluation of the AI-CT and CBCT images was conducted by comparing the measurements of the tumor shadow size and volume for both image types. This evaluation was performed in the iPlan treatment planning system. Stationary and moving lesions in the AI-CT and CBCT images were contoured using a window/level setting of 1000/-500. When the blurring of the tumor shadow was strong, the window values/levels were adjusted manually.

The discrepancies (residual errors) from manual image matching for the AI-CT and CBCT images, *D*_*match*_, were subsequently determined by evaluating the performance of five independent operators (two medical physicists and three radiation technologists), and all matching results were confirmed by two radiation oncologists. The manual image matching was performed in the ExacTrac system. The procedure of manual image matching was based on matching the tumor shadow center of gravity, and then confirmed by the agreement of the ITV contour and CBCT image. Prior to initiating the research, all operators were familiar with and proficient in the performance of the procedure. Fig 2e and 2f shows samples of the AI-CT and the CBCT images, respectively.

### Overall geometric accuracy of the Vero4DRT IGRT system for SBRT

The overall geometric accuracy was determined by separating the overall deviation and overall variation. The overall deviation, *D*_*all*_, is defined as

$$D_{all}=\sqrt{{\left(D_{CBCT-MV}\right)}^{2}+{\left(D_{match}\right)}^{2}},$$ (5)

where *D*_*CBCT-MV*_ and *D*_*match*_ represents the discrepancy between the CBCT image center and MV beam isocenter and the discrepancy from manual image matching, respectively, as described in the previous sections. In accordance with the uncertainties budget (ISO 1995), overall variation, *U*_*all*_, is defined as [19, 20]

$$U_{all}=2\times \sqrt{{\left(\frac{u_{1}}{\sqrt{3}}\right)}^{2}+{\left(\frac{u_{2}}{\sqrt{3}}\right)}^{2}+{\left(\frac{u_{3}}{\sqrt{3}}\right)}^{2}+{\left(\frac{u_{4}}{1.0}\right)}^{2}},$$ (6)

where *u*1, *u*2, *u*3, and *u*4 represent the variations in gantry rotation, ring rotation, CBCT scan parameter setting, and image matching, respectively. The variation value computed from several measurements was normally distributed. In this case, the uncertainty was divided by a factor of 1.0 (Type A evaluation of standard uncertainty). In contrast, the variation value was divided by a factor of √3, when the maximum value of the variation was known, or the limit value was used (Type B evaluation of standard uncertainty). As for *D*_*CBCT-MV*_ and *D*_*match*_, the vector lengths were calculated using the following formula:$v=\sqrt{\left(x^{2}+y^{2}+z^{2}\right)}$. The vector length of *D*_*all*_ was then determined using Eq (5).

## Results

### Coincidence of the CBCT center and MV beam isocenter

**Fig 3:**
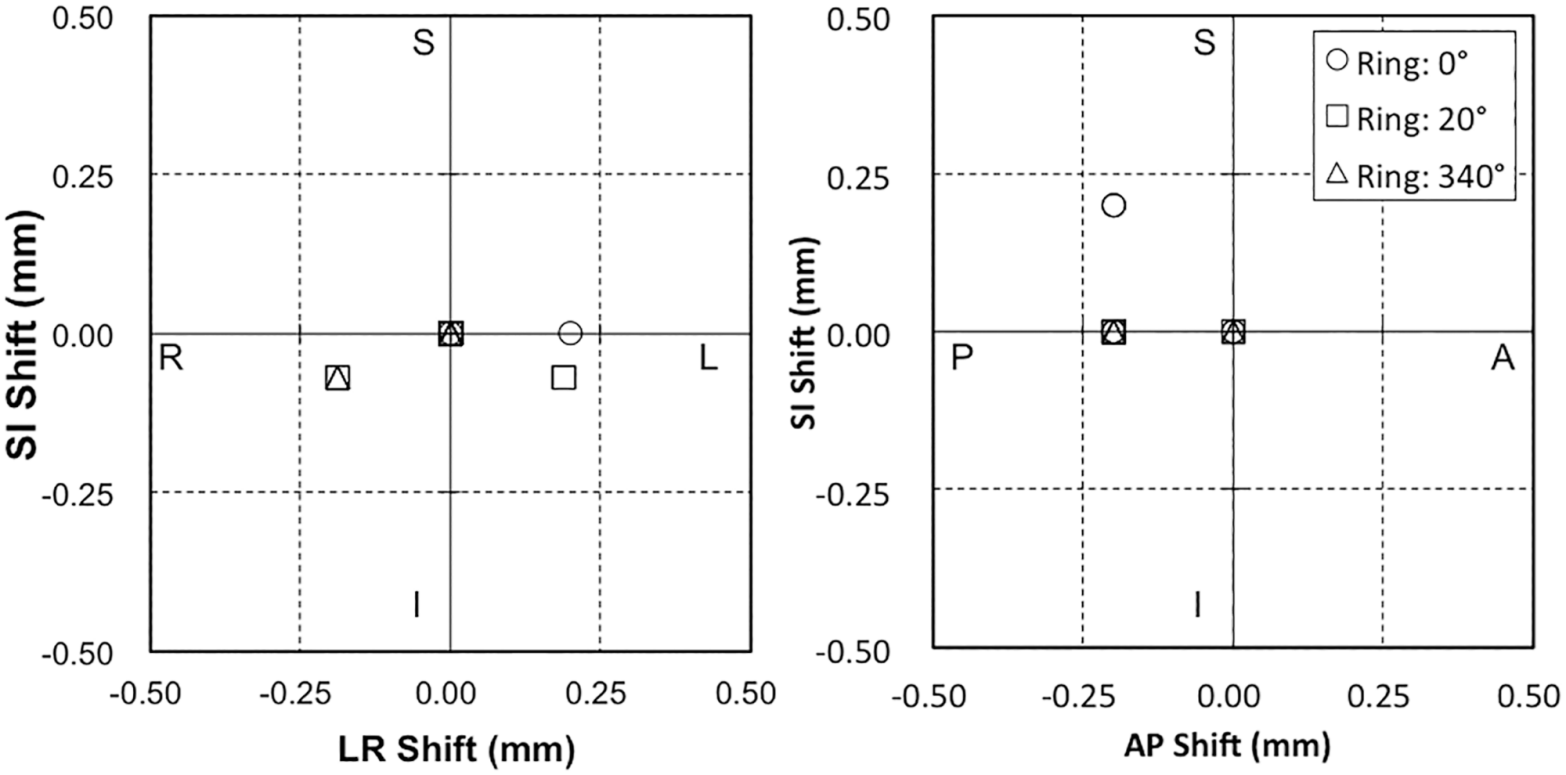
Spatial discrepancies between the steel ball and MV X-ray beam for gantry rotation values of 0°, 90°, 180°, and 270°, and ring rotation values of 0°, 20°, and 340°.

The coincidence of the steel ball and the MV beam were estimated (Fig 3). The average directional data values were LR = −0.02 ± 0.04 mm, AP = −0.15 ± 0.06 mm, and SI = −0.02 ± 0.03 mm. Further, the largest position errors by direction were LR = 0.20 mm, AP = 0.20 mm, and SI = 0.20 mm.

**Fig 4:**
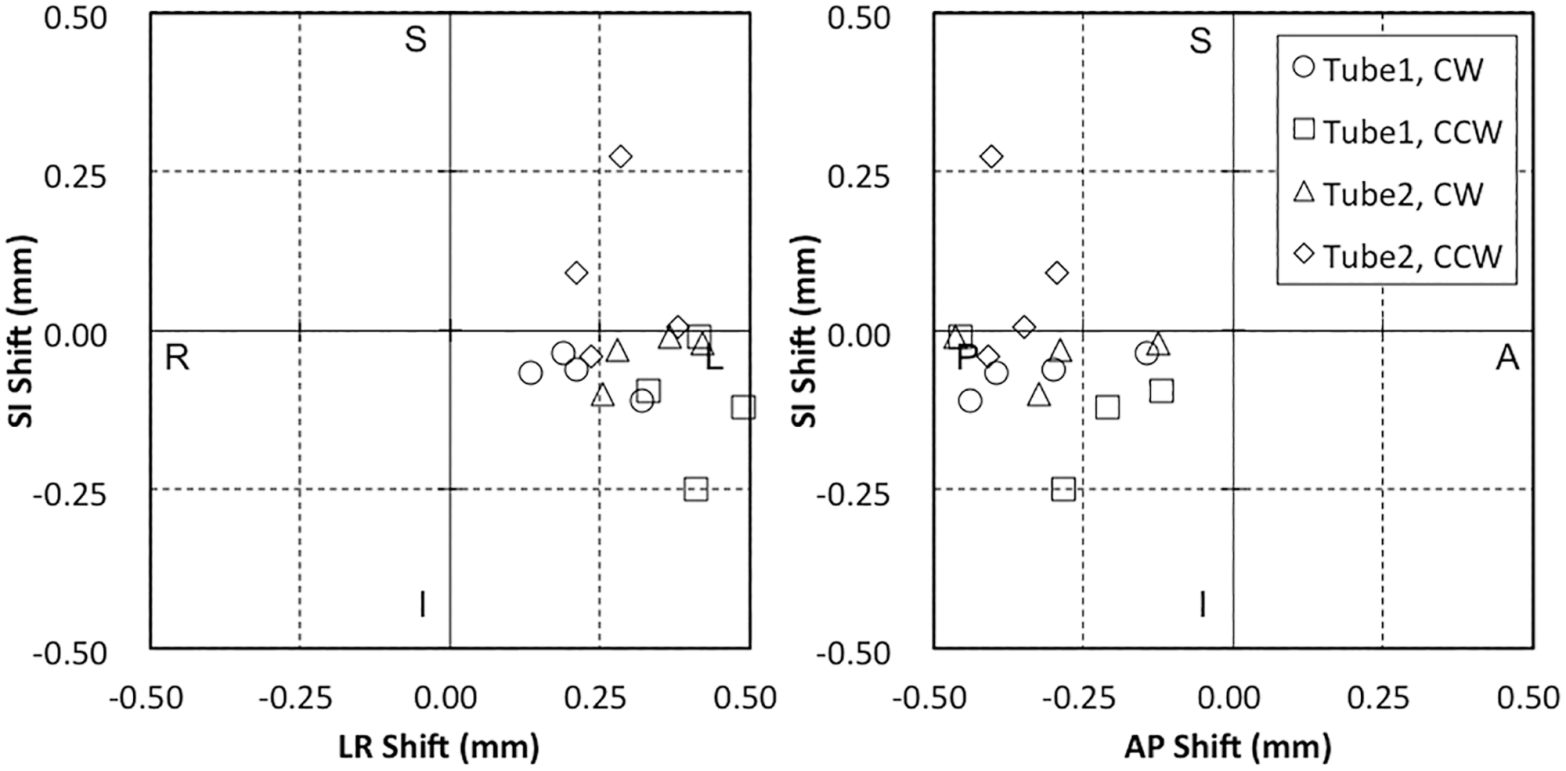
Spatial discrepancies between the steel ball and CBCT corresponding to changes in CBCT imaging parameters between Tube 1 and Tube 2, or switching rotation direction between clockwise (CW) or counterclockwise (CCW).

Fig 4 represents the results for coincidence of the steel ball and the CBCT center. The average directional data values were LR = 0.31 ± 0.05 mm, AP = −0.31 ± 0.08 mm, and SI = −0.04 ± 0.03 mm. The largest position errors were LR = 0.49 mm, AP = 0.12 mm, and SI = 0.28 mm.

**Fig 5:**
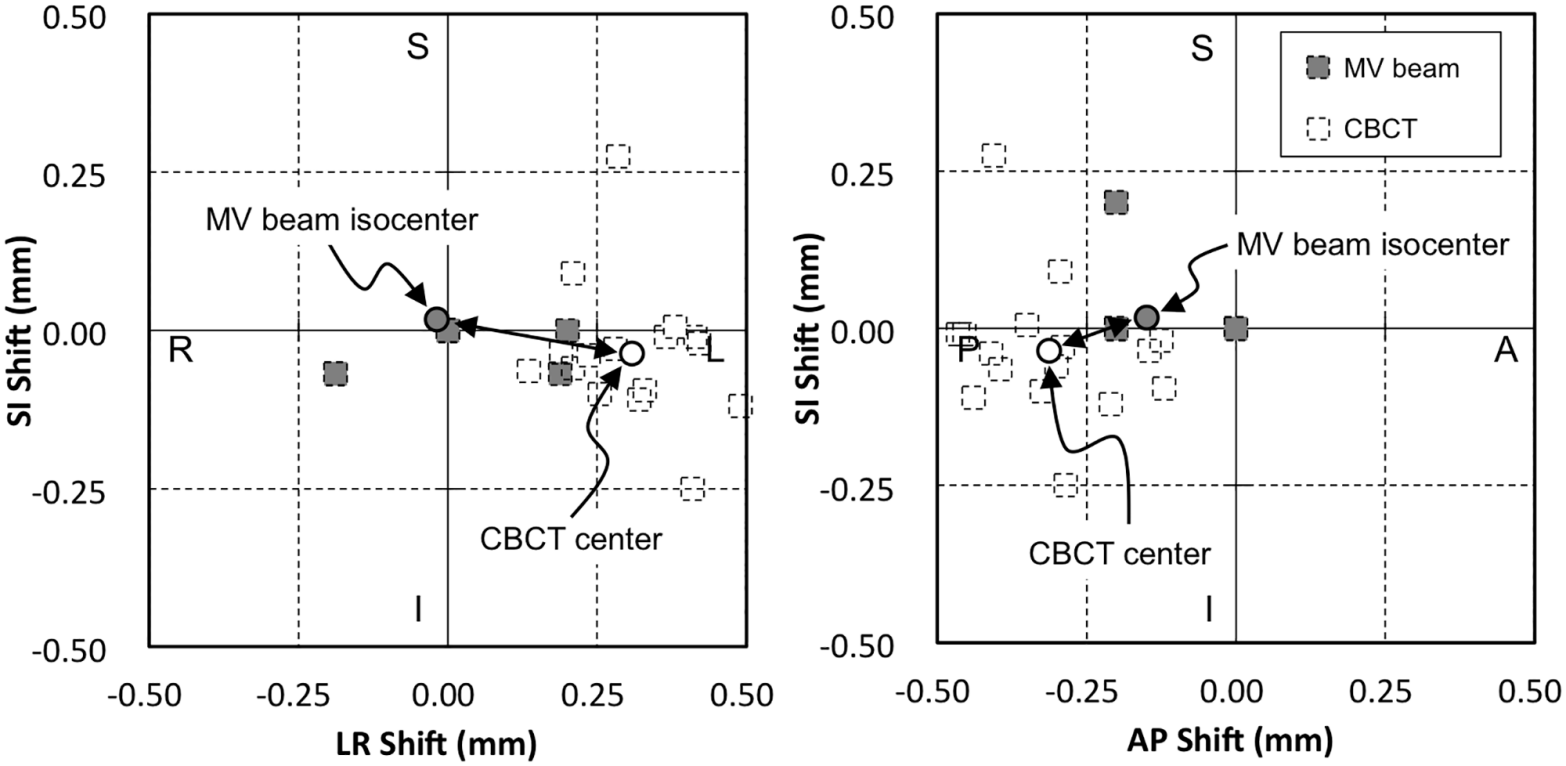
Spatial discrepancy between the MV X-ray beam isocenter and CBCT center. The center of the MV beam and CBCT were calculated from the mean discrepancy between the MV beam or CBCT and the steel ball, respectively. The dotted line markings show the original data points of these discrepancies. The double arrows indicate the mean discrepancy of the MV beam isocenter and CBCT center.

Fig 5 shows the coincidence of the MV beam isocenter and the CBCT center. The mean discrepancies of *D*_*CBCT-MV*_ were LR = 0.33 ± 0.09 mm, AP = −0.16 ± 0.07 mm, and SI = −0.05 ± 0.04 mm. The largest position errors were LR = 0.55 mm, AP = 0.27 mm, and SI = 0.08 mm.

### Manual image matching using blurred images

Table 1 lists the discrepancies in the theoretical values of tumor shadow diameters for the AI-CT and CBCT images. For both image types, the largest variance was 0.8 mm, which represented a high level of consistency. The uniformity of tumor shadow volumes between the theoretical volumes and the volumes in AI-CT and CBCT images are shown in Table 2. The disagreement in the values associated with volume increased with large motion amplitude of the tumor. Likewise, a similar tendency was apparent in the results of the AI-CT and CBCT.

**Table 1:** Tumor shadow consistency measurements showing a comparison between the theoretical tumor shadow size and averaged intensity (AI) and cone-beam computed tomography (CBCT) images.

Amplitude	Period	Large simulated tumor (30 mmΦ)						Small simulated tumor (20 mma)					
		AI-CT image			CBCT image			AI-CT image			CBCT image		
		LR	AP	SI	LR	AP	SI	LR	AP	SI	LR	AP	SI
(mm)	(s)	(mm)						(mm)					
0	0	0.4	0.3	1.3	0.4	0.4	1.1	0.4	0.0	1.0	0.1	0.1	0.9
15	2	0.4	0.5	-1.0	0.1	0.1	-1.0	0.1	0.0	-0.5	0.4	0.2	-0.9
15	4	0.4	0.1	-0.8	0.1	0.1	-1.0	0.0	0.1	-1.4	0.0	0.3	-1.0
30	2	0.0	0.1	-0.9	0.1	0.1	-1.6	0.2	0.0	-0.9	0.0	0.2	-0.8
30	4	0.2	0.1	-1.5	0.0	0.0	-0.7	0.0	0.1	-1.1	0.3	0.2	-1.2

The theoretical tumor shadow sizes were calculated from both the tumor size and the amplitude of the tumor motion.

*Abbreviations*: LR = left-right; AP = anterior-posterior; SI = superior-inferior.

**Table 2:** The consistency of tumor shadow volumes between the theoretical volumes and the volumes in averaged intensity (AI)-CT and CBCT images.

Amplitude	Period	Large simulated tumor		Small simulated tumor	
		AI-CT	CBCT	AI-CT	CBCT
(mm)	(s)	(%)	(%)	(%)	(%)
0	0	4.5	6.2	5.8	7.4
15	2	-6.2	-4.2	-6.0	-5.0
15	4	-9.2	-7.1	-6.3	-9.6
30	2	-10.6	-9.3	-11.7	-12.6
30	4	-10.1	-7.3	-10.5	-11.9

The theoretical volumes were calculated from both the tumor size and the amplitude of the tumor motion.

**Fig 6:**
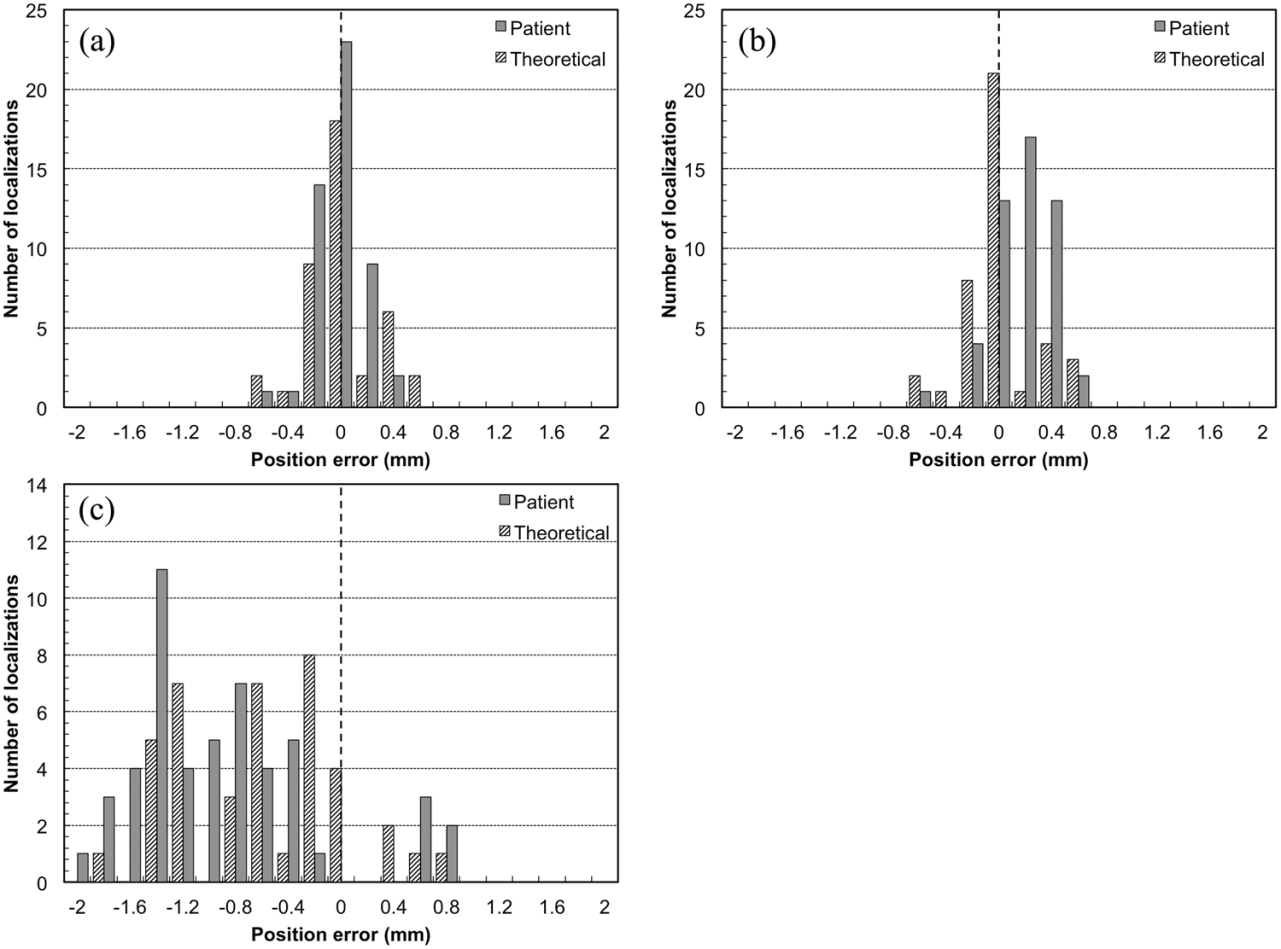
Histogram illustrating discrepancies in manual image matching with respect to image blurring for (a) the left-right (LR) direction, (b) the anterior-posterior (AP) direction, and (c) the superior-inferior (SI) direction.

Fig 6 shows a histogram that illustrates the residual manual image matching error for the five independent operators. For the theoretical breathing patterns study, the mean residual error after target localization using the LR, SI, and AP directions were -0.01 ± 0.27, −0.62 ± 0.63, and -0.02 ± 0.28 mm, respectively. For the patient breathing patterns study, the mean residual error after target localization using the LR, SI, and AP directions were -0.05 ± 0.18, −0.93 ± 0.70, and -0.14 ± 0.21 mm, respectively (Table 3). The SI direction exhibited the largest average variance and standard deviation (SD) in the directional data value.

**Table 3:** Mean manual matching error and standard deviation using AI-CT images and CBCT images, which were obtained by five independent operators.

Direction	Theoretical breathing patterns	Patient breathing patterns	All tests
	(mm)	(mm)	(mm)
L(+)/R(−)	−0.01 ± 0.27	−0.05 ± 0.18	−0.03 ± 0.22
S(+)/I(−)	−0.62 ± 0.63	−0.93 ± 0.77	−0.79 ± 0.68
A(+)/P(−)	−0.02 ± 0.28	0.14 ± 0.21	0.07 ± 0.25

### Overall geometric accuracy of the Vero4DRT IGRT system for SBRT

Table 4 summarizes the overall deviation calculated using Eq (5). The overall deviations for the LR, SI, and AP directions were 0.33, 0.79, and 0.17 mm, respectively. The vector length of the overall deviation was 1.07 mm. The overall variation calculated using Eq (6) is summarized in Table 5. The overall variations for the LR, SI, and AP directions were 0.84, 1.43, and 0.60 mm, respectively. The overall variation of the vector length was 1.23 mm.

**Table 4:** Alignments conducted between the MV X-ray beam isocenter and the CBCT image-guidance system, and a comprehensive evaluation of the manual image matching accuracy, with an overall deviation of *D*_*all*_.

Direction	*D*_*CBCT-MV*_	*D*_*match*_	*D*_*all*_
L(+)/R(−) (mm)	0.33	−0.03	0.33
S(+)/I(−) (mm)	−0.05	−0.79	0.79
A(+)/P(−) (mm)	−0.16	0.07	0.17
Vector length (mm)	0.41	0.99	1.07

**Table 5:** Alignments conducted between the MV X-ray beam isocenter and the CBCT image-guidance system, and a comprehensive evaluation of the manual image matching accuracy.

Source of uncertainty	Gantry rotation	Ring rotation	CBCT setting	CBCT registration	*U*_*all*_
	*u*_*1*_	*u*_*2*_	*u*_*3*_	*u*_*4*_	
Divided factor	$\sqrt{3}$	$\sqrt{3}$	$\sqrt{3}$	1	
LR (mm)	0.20	0.20	0.55	0.22	0.84
SI (mm)	0.20	0.20	0.27	0.68	1.43
AP (mm)	0.20	0.20	0.08	0.25	0.60
Vector length (mm)	0.28	0.28	0.56	0.47	1.23

The alignments exhibited an overall variation of *U*_*all*_ (95% confidence interval).

## Discussion

When the gantry and ring were rotated, the greatest variation in MV X-ray beam axial position was 0.20 mm. The pixel resolution of EPID was 0.18 × 0.18 mm^2^, and error values less than 0.2 mm were rounded off according to the specification of the Daily Check tool. However, the result of the Daily Check tool, based on an EPID, corresponded to 0.02 ± 0.11 mm using the Winston-Lutz tests on radiochromic films. Prior to initiation of the research, we confirmed the high reliability of the Daily Check tool. Miyabe *et al*. reported the positioning accuracy of the Vero4DRT, and determined that the largest variation in beam axial position from the isocenter was less than 0.5 mm [16]. Thus, the results of the current study were largely in agreement with the previous findings. Depuydt *et al*. reported on the mechanical performance of the isocenter with the Vero4DRT using the star shot films evaluation technique [15]. The results indicated that the isocenter radius for gantry rotation differed with the time of year the equipment was installed, with resulting sizes of 0.12 and 0.25 mm for the equipment installed in 2011, and approximately 0.4 mm with the equipment installed in 2010. Therefore, the slight difference apparent between the results of the current study and the previously reported values [16] may have been caused by the time of equipment installation, as described earlier [15]. Additionally, Weiliang *et al*. reported that the radiation field center wobbled in the SI direction by 0.82 mm from the gantry angle of 0° to 180° for the Varian Trilogy linear accelerator [21], which was attributed to the gantry tilt and/or sagging. The positional discrepancies of radiation field in the SI direction are also reported as 0.69 mm and 0.55 mm for the BrainLAB Novalis and the Varian TrueBeam, respectively [19, 22]. When compared with those results, the Vero4DRT exhibited equal or superior alignment accuracy. We observed the effectiveness of the lock-on system for the gimbal on the Vero4DRT.

The current examination detected a basic reference point discrepancy between the MV X-ray beam and the CBCT value of less than 0.5 mm. The pixel size in the CBCT images produced by the Vero4DRT was 0.39 mm, and the discrepancy detected by this examination was limited to within two pixels. Our results also largely agreed with the results of Miyabe *et al*. [16]. The individual specificity of the geometric accuracy of the Vero4DRT has not yet been evaluated. Based on the results reported, it seems reasonable to suppose that the individual specificity of the geometric accuracy of the Vero4DRT is small. Bissonnette *et al*. reported a 95% confidence interval within ± 0.5 mm and a 99% confidence interval within ± 2 mm with respect to the Synergy linac model [23]. Our examination only included data collected following four days of evaluation, while the examination by Bissonnette *et al*. included data collected over a three-year evaluation period. However, we believe that our data exhibited equivalent or greater levels of geometric accuracy when compared with those of Bissonnette *et al*. Additionally, the coincidence of the CBCT center and the MV beam isocenter were within the management threshold value of ± 1 mm for AAPM TG-142 and TG-179 [24, 25].

Our study was conducted based on several valuable points extracted from previous publications. However, most of the previous studies were performed with the stable phantom and/or automatic image matching [10, 11, 16]. Conversely, we evaluated the accuracy of manual image matching using blurred images based on the observations of five independent operators. Further, this study addressed the essential issue of accuracy in the setup correction for the SBRT. We believe that the accuracy of the currently available automatic image matching system (i.e. 3D rigid registration techniques) was not sufficient for image matching using free-breathing CBCT. The primary cause of the problems would be the different image quality between the simulation CT with CBCT images and the anatomical changes of the inter-fraction (e.g. correlation of the position of a pulmonic field and tumor). When performing automatic image matching in the whole area of the CBCT, the tumor shadow did not match in many cases because the fusion results depended on the bigger internal organs (e.g. the pulmonic field). On the other hand, the region of automatic image matching could only be selected near a tumor shadow. A problem associated with automatic image matching was the low level of apparent contrast between the lung and the tumor shadow, and the spherical tumor shadow, which caused positional and rotational errors. Regarding SBRT, the tumor localization based on the CBCT was thus performed through manual image matching. We therefore estimated the accuracy of manual image matching using the motion phantom. The overall geometric accuracy was then described accordingly by the coincidence of the CBCT center and MV beam isocenter, as well as by the accuracy of the manual image matching.

The tumor shadow diameters and volumes from both the AI-CT and the CBCT images were in good agreement. Clements *et al*. described the mismatch in volume between the tumor shadow on 4DCT MIP and free-breathing CBCT [26]. In these results, the tumor shadow size of CBCT became smaller than the 4DCT MIP, depending on the motion amplitude. Conversely, in the current study, the volumes of the AI-CT and CBCT images exhibited the same reduction tendency. Based on these results, we believe that it is possible to utilize the AI-CT and CBCT images as standard for image matching.

The CBCT images acquired using the Vero4DRT had some basic limitations with regard to image quality. The limitations were caused by the small FOV (215 mm), incomplete gantry rotation (200°), and disabled bowtie filters. We have requested the manufacturer to make qualitative improvements to the CBCT images. These device-specific limitations may have affected the results of manual image matching.

Deviation and variation values, as determined by five independent operators conducting manual image matching, were the largest in the SI direction, which was attributed to movement during respiration. On the basis of the results shown in Tables 4 and 5, it is apparent that the deviations and variations in manual image matching in the SI direction contributed to a large part of the overall geometric accuracy. We hypothesized that CT slice thickness was one of the factors affecting the accuracy of manual image matching, and it may therefore be possible to improve the accuracy using a thin-slice reconstructed CT (e.g., 1.25-mm-thick slices). Furthermore, we are certain that the main factor affecting the accuracy of manual image matching was the image quality of the free-breathing CBCT. To overcome this limitation, Zhang *et al*. reported a superior method to correct respiratory motion artifact in CBCT scans, using a patient-specific motion model derived from a respiration-correlated CT image set [27, 28]. To achieve the same aim, Kincaid *et al*. evaluated the respiration-gated CBCT technique, which was programmed such that gantry rotation and kV imaging acquisition occur with a gate around end expiration [29]. After that, the outcomes of the prospective study comprising a large group of lung cancer patients showed improvement in the accuracy of target localization on the respiration motion-corrected CBCT [30]. On the other hand, 4D-CBCT has been recently commercialized and performed using a volumetric 4D image-guided workflow [31]. Unfortunately, the Vero4DRT can only perform free-breathing CBCT, because this system is not currently equipped with novel techniques to reduce motion artifacts. To further enhance the accuracy of target localization, motion correction methods in CBCT are highly effective.

This study had some limitations. First, the results are only valid for phantom use. Actual tumors have a complex shape and size and inter- and intrafractional changes. This may not represent the actual accuracy of manual image matching. Another limitation is the artifact from the small steel markers on the CBCT images. We employed small steel markers for higher accuracy in position verification. Further research, especially prospective studies that include the patient CBCT images with using a precise criterion standard for comparison, is therefore necessary to assess complex cases. The gated CBCT at end expiration served as the criterion standard in the study conducted by Dzyubak *et al*. [30].

## Conclusions

We verified both the geometric accuracy of the CBCT image-guidance system on the Vero4DRT, and the inter-observer variation of image matching between the AI-CT and CBCT images. The overall geometric accuracy of the Vero4DRT using CBCT was at 1.07 ± 1.23 mm (2 SD). We were able to ensure a sufficient level of geometric accuracy using the free-breathing CBCT and the image-guidance system mounted on the Vero4DRT. However, the inter-observer variation of image matching substantially affected the overall geometric accuracy. Misalignment of image matching will cause significant systematic localization errors. Therefore, when using the free-breathing CBCT images, careful consideration of image matching is especially important.
